# Synaptic Inhibition in Avian Interaural Level Difference Sound Localizing Neurons

**DOI:** 10.1523/ENEURO.0309-16.2016

**Published:** 2016-12-20

**Authors:** Rebecca J. Curry, Yong Lu

**Affiliations:** 1 Department of Anatomy and Neurobiology, College of Medicine, Northeast Ohio Medical University, Rootstown, Ohio 44272; 2 School of Biomedical Sciences, Kent State University, Kent, Ohio 44240

**Keywords:** dorsal nucleus of the lateral lemniscus, GABA_A_ receptor, interaural level difference, reversal potential, synaptic inhibition

## Abstract

Synaptic inhibition plays a fundamental role in the neural computation of the interaural level difference (ILD), an important cue for the localization of high-frequency sound. Here, we studied the inhibitory synaptic currents in the chicken posterior portion of the dorsal nucleus of the lateral lemniscus (LLDp), the first binaural level difference encoder of the avian auditory pathway. Using whole-cell recordings in brain slices, we provide the first evidence confirming a monosynaptic inhibition driven by direct electrical and chemical stimulation of the contralateral LLDp, establishing the reciprocal inhibitory connection between the two LLDps, a long-standing assumption in the field. This inhibition was largely mediated by GABA_A_ receptors; however, functional glycine receptors were also identified. The reversal potential for the Cl^−^ channels measured with gramicidin-perforated patch recordings was hyperpolarizing (−88 mV), corresponding to a low intracellular Cl^−^ concentration (5.2 mm). Pharmacological manipulations of KCC2 (outwardly Cl^−^ transporter) activity demonstrate that LLDp neurons can maintain a low intracellular Cl^−^ concentration under a high Cl^−^ load, allowing for the maintenance of hyperpolarizing inhibition. We further demonstrate that hyperpolarizing inhibition was more effective at regulating cellular excitability than depolarizing inhibition in LLDp neurons.

## Significance Statement

Sensory processing performed by distinct neural circuits requires proper synaptic inhibitory inputs. Properties of synaptic inhibition, such as transmitter types and ionic mechanisms of synaptic responses, vary among different neural circuits. Here, we provide the first physiological evidence demonstrating direct inhibitory connections between two avian auditory brainstem nuclei that encode information for sound localization using interaural level difference as a cue. We have characterized the physiological and pharmacological properties of this synaptic inhibition and demonstrate the role of effective hyperpolarizing inhibition in the circuit.

## Introduction

The location of sound is not encoded by the sensory epithelium but is computed centrally in the auditory brainstem. Although tympanic auditory organs evolved independently across tetrapod groups (for review, see Grothe et al., 2010; Carr and Christensen-Dalsgaard, 2016), sound localization circuitry shows similarities across species and relies on the same types of acoustical information to form neural representations of auditory space. For sounds in the horizontal plane, these circuits rely on binaural cues such as interaural time differences (ITDs) and interaural level differences (ILD). ITDs are first encoded by the medial superior olive in mammals and the nucleus laminaris (NL) in birds, while ILDs are first encoded in the mammalian lateral superior olive (LSO) and the avian posterior portion of the dorsal nucleus of the lateral lemniscus [LLDp (formerly VLVp, nucleus ventralis lemnisci lateralis pars posterior; Mogdans and Knudsen, 1994)]. Much is known of the synaptic properties that enable the transformation of auditory information in the ITD pathway of birds and mammals, and in the mammalian ILD pathway (for review, see Kubke and Carr, 2005; Joris and Yin, 2007; Grothe et al., 2010; Burger et al., 2011). However, the synaptic properties of the avian ILD pathway remain poorly understood.

Synaptic inhibition mediated by glycine and GABA plays important roles in ITD and ILD coding, and distinct differences in synaptic inhibitory mechanisms exist between the mammalian and avian systems (for review, see Grothe et al., 2010; Grothe and Pecka, 2014). For ITD coding, precisely timed fast glycinergic inhibition is a hallmark of the mammalian circuit, whereas slow and sustained GABAergic inhibition is a prominent feature of the avian circuit, although both transmitter components exist in each circuit (Code and Rubel, 1989; Wu and Kelly, 1992; Stange et al., 2013; Fischl and Burger, 2014). Another important difference is the inhibition in the mammalian circuit switches from depolarizing to hyperpolarizing during development (Kandler and Friauf, 1995; Ehrlich et al., 1999), whereas inhibition in the avian ITD circuit remains depolarizing (Hyson et al., 1995; Lu and Trussell, 2001; Monsivais and Rubel, 2001; Tang et al., 2009). For ILD coding in the LSO, the primary inhibitory input driven by the contralateral ear is glycinergic and forms the basis of the ILD coding along with the ipsilateral excitation. The glycinergic inputs to the LSO are hyperpolarizing and specialized with short time constants and fast receptor kinetics, ensuring registry in timing between synaptic excitation and inhibition, an essential requirement for ILD coding (for review, see Tollin, 2003). Meanwhile, high spiking activity in LSO neurons leads to the release of GABA from the same neurons, which regulates excitation and inhibition via retrograde activation of GABA_B_ receptors on the presynaptic terminals, providing an activity-dependent regulatory mechanism (Magnusson et al., 2008).

In the avian ILD circuit, LLDp neurons receive excitatory input from the contralateral cochlear nucleus angularis (NA; Conlee and Parks, 1986; Takahashi and Konishi, 1988) and receive inhibitory input primarily from the other LLDp, which is driven by excitatory input originating from the ipsilateral NA (Manley et al., 1988; Takahashi and Keller, 1992). Of particular interest are the cellular mechanisms of synaptic inhibition in this circuitry, as it is unknown what cells in the LLDp give rise to the inhibition, whether both glycine and GABA underlie the inhibition, whether the inhibition is hyperpolarizing or depolarizing, and whether the cellular specializations are consistent with our understanding of ILD coding constraints. Additionally, while there is a known role for tonic inhibition in the LLDp providing gain modulation to enhance the reliability of envelope locking in spectrotemporal processing (Steinberg et al., 2013), the cellular physiology and pharmacology of the inhibition from the reciprocal inputs has not been well addressed. Most importantly, direct physiological evidence demonstrating reciprocal inhibitory connections between the two LLDps is lacking. To address these issues, we determined the physiological properties of the synaptic inhibition in LLDp neurons using whole-cell and perforated patch-clamp recordings from chicken brainstem slices combined with pharmacological manipulation and immunohistochemistry.

## Materials and Methods

### Slice preparation and *in vitro* whole-cell recordings

Brainstem slices (300 μm in thickness) were prepared from white leghorn chick embryos [embryonic day 17 (E17) to E19] of both sexes, as described previously (Tang et al., 2011). While the majority of LLDp studies have historically used barn owls as the avian model, the ILD circuitry appears to be conserved anatomically between avian species (Kubke and Carr, 2000; Wild et al., 2010). One *in vivo* study has shown that the chicken LLDp is able to encode ILD (Sato et al., 2010), so we used the chick as our animal model. The selected age ranges (E17–E19) represent the relative maturation of cellular properties in avian auditory brainstem neurons (Gao and Lu, 2008), which is developmentally equivalent to that of postnatal day 17 (P17) to P19 rodents. The warm (35**°**C) artificial CSF (ACSF) used for dissecting and slicing the brain tissue contained the following (in mm): 250 glycerol, 3 KCl, 1.2 KH_2_PO_4_, 20 NaHCO_3_, 3 HEPES, 1.2 CaCl_2_, 5 MgCl_2_, and 10 glucose, pH 7.4 (when gassed with 95% O_2_ and 5% CO_2_). The procedures have been approved by the Institutional Animal Care and Use Committee at Northeast Ohio Medical University and were performed in accordance with National Institutes of Health policies on animal use. Slices were incubated in an interface chamber at 34–36°C for >1 h in normal ACSF containing the following (in mm): 130 NaCl, 26 NaHCO_3_, 3 KCl, 3 CaCl_2_, 1 MgCl_2_, 1.25 NaH_2_PO_4_, and 10 glucose, pH 7.4. For recording, slices were transferred to a 0.5 ml chamber mounted on a Zeiss Axioskop 2 FS Plus microscope with a 40× water-immersion objective and infrared differential interference contrast optics. The chamber was continuously superfused with ACSF (2–5 ml/min) by gravity.

Patch pipettes were drawn on a PP-830 Microelectrode Puller (Narishige) to a 1–2 μm tip diameter using borosilicate glass micropipettes (inner diameter, 0.84 mm; outer diameter, 1.5 mm; World Precision Instruments). The electrodes had resistances between 3 and 6 MΩ when filled with a solution containing the following (in mm): 125 K-gluconate, 5 Na-gluconate, 10 NaCl, 5 EGTA, 10 HEPES(K), 1 CaCl_2_, 1 MgCl_2_, 4 ATP-Mg, 0.48 GTP-Na, pH 7.3 (adjusted with KOH and osmolarity between 280 and 290 mOsm/L). Therefore, a Cl^−^ concentration of 14 mm in the internal solution was used in whole-cell recording (WCR). Biocytin (0.1%) was added to the internal solution to reveal cell morphology and location. The liquid junction potential was 13 mV, and data were corrected accordingly. Voltage-clamp and current-clamp experiments were performed with an AxoPatch 200B and an AxoClamp 2B amplifier, respectively (Molecular Devices). Recordings were performed under warm temperatures (34–36°C). Voltage-clamp recordings were obtained at a holding potential of −73 mV, and current-clamp recordings were obtained at the resting membrane potential (RMP). Data were low-pass filtered at 3–10 kHz and digitized with a Data Acquisition Interface ITC-18 (InstruTech) at 50 kHz. Recording protocols were written and run using the acquisition and analysis software AxoGraph X (AxoGraph Scientific).

All chemicals were purchased from Sigma-Aldrich except for gabazine (catalog #SR 95531), which was obtained from Tocris Bioscience, and DNQX, which was obtained from Abcam.

### Electrical stimulation experiments

Extracellular stimulation was performed using concentric bipolar electrodes with a tip core diameter of 127 μm (World Precision Instruments). The stimulating electrodes were placed using a NMN-25 Micromanipulator (Narishige) and were positioned medial to the LLDp (medial stimulation) or directly in the contralateral LLDp (contralateral stimulation) to activate the inhibitory afferent fibers. Such placement of the stimulating electrodes could evoke one of the following responses in a particular recorded LLDp neuron under normal ACSF perfusion: IPSC only or IPSC plus EPSC. The observation of the responses in the second category was rarely seen during contralateral LLDp stimulation. IPSCs and IPSPs were isolated pharmacologically with an antagonist for AMPARs (50 μm DNQX) in all stimulation experiments. NMDARs were not blocked in experiments where the membrane potential was held at levels more negative than −20 mV, with presumably minimal activation of NMDARs because of the Mg^2+^ block of the receptors. For gramicidin experiments where the membrane holding potential was stepped to levels more depolarized than −20 mV, APV (25 μm) was also included to block the NMDAR-mediated current. GABAergic and glycinergic currents and potentials were confirmed by bath application of the GABA_A_ receptor (GABA_A_R) antagonist gabazine (10 μm) and glycine receptor antagonist strychnine (1 μm), respectively.

### Agonist puff experiments

Puff application of muscimol (10 µm) and glycine (500 µm) was used to determine the presence of GABA_A_ and glycine receptors, respectively. Puff electrodes were drawn on a PP-830 Microelectrode Puller (Narishige) to a 5 μm tip diameter using borosilicate glass micropipettes (VWR Scientific) and filled with ACSF containing the appropriate agonist, at pH 7.4. To elicit the maximal response, the puff electrode was placed 20–30 μm from the cell body, and the solution was pressure ejected at 5–10 psi for 25–50 ms using a Picospritzer. Experiments were recorded in the presence of DNQX (50 μm), and receptor activation was confirmed by bath application of the respective antagonists gabazine (10 μm) and strychnine (1 μm).

### Gramicidin-perforated patch recordings

Gramicidin-perforated patch recordings allow electrical access to neurons without disturbing the native Cl^−^ concentration (Kyrozis and Reichling, 1995). The intracellular pipette solution contained the following (in mm): 140 KCl, 5 EGTA, 10 HEPES, 2 MgCl_2_, and 0.5 CaCl_2_, and pH was adjusted with KOH to 7.4, and osmolarity was measured at between 280 and 290 mOsm/L. The use of a high Cl^−^ concentration (145 mm, equal to the extracellular Cl^−^ concentration) allowed us to detect when a perforated patch broke into whole-cell mode, which would result in a Cl^−^ equilibrium potential at ∼0 mV. The tip of the patch pipette was filled with the high Cl^−^ solution and then backfilled by syringe with the same solution containing gramicidin dissolved in DMSO with a final concentration of 5–25 μg/ml. The liquid junction potential was 5 mV, and data were corrected accordingly. The initial series resistance after gigaohm seal formation exceeded 100 MΩ, but could decline down to 20–40 MΩ within 30 min, at which time data acquisition began. Recordings were discarded if the perforated patch ruptured, as indicated by an abrupt drop in series resistance and the measure of IPSC reversal potential (E_rev_) at ∼0 mV.

### Anatomical experiments

**Figure 1.**
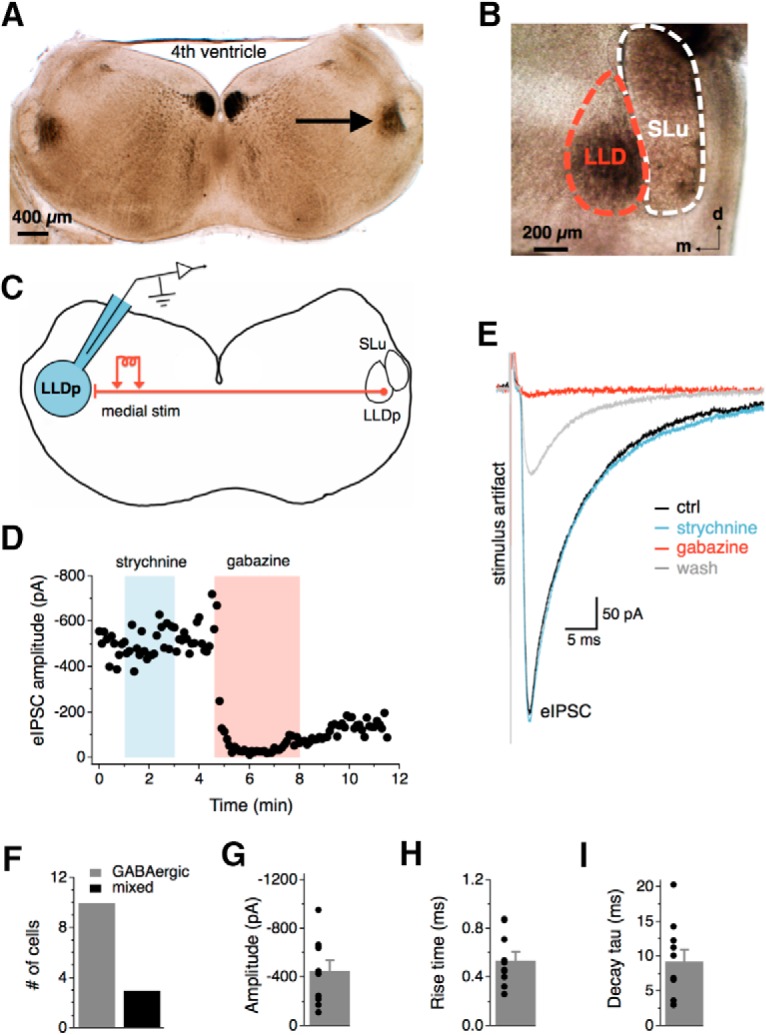
Synaptic inhibition in the LLDp is largely GABAergic. ***A***, The LLDp (arrow) is located laterally within a 300-μm-thick coronal brainstem slice with ∼4 mm between the two LLDps. ***B***, The LLDp is readily identifiable as a heavily myelinated nucleus medial and slightly ventral to the semilunar nucleus (SLu) in fresh tissue slices. ***C***, Schematic of the experimental setup highlighting ipsilateral recording site (blue, left), and medial electrical stimulation of fibers projecting from the contralateral LLDp. ***D***, Bath application of gabazine (10 μm), a GABA_A_ receptor antagonist, abolished the eIPSC, whereas the eIPSC amplitude was not affected by strychnine (1 μm), a glycine receptor antagonist. ***E***, Averaged traces of the eIPSC during control (black), strychnine (1 μm, blue), gabazine (10 μm, red), and wash (gray). ***F***, In the majority of cells, eIPSCs were abolished by gabazine alone (*n* = 10), although occasionally an additional weak strychnine component was observed (*n* = 3). ***G–I***, Population data of eIPSC amplitude, 20–80% rise time, and decay time constant (tau) for GABAergic eIPSCs (*n* = 10). For this and subsequent figures, mean ± SEM values are shown. d, dorsal; m, medial; ctrl, Control; stim, stimulation.

The LLD was easily recognized as a heavily myelinated ovoid region at the lateral margin of coronal brainstem slices medial and ventral to the semilunar nucleus in fresh tissue slices (Fig. 1*A*,*B*). The border of the LLDp was distinguished by a medial lamina, such that cells were sampled from the lateral portion of the LLD, which corresponds to the LLDp (Heil and Scheich, 1986). Slices containing biocytin-filled cells were fixed in 4% paraformaldehyde (PFA) in 0.1 m phosphate buffer overnight (O/N) and were processed with a Vectastain ABC Elite Kit (Vector Laboratories). Neurons recorded from outside the designated boundaries were discarded. For Nissl-stained tissue (P2–P9; *n* = 2), 50-μm-thick slices were mounted and dried, stained with cresyl violet, dehydrated, permanently coverslipped with Permount (Fisher Scientific), and photographed under standard bright-field illumination.

The reagents for immunostaining were purchased from Sigma-Aldrich, unless otherwise indicated. To study the distribution of inhibitory neurons, the expression of glutamic acid decarboxylase 65/67 (GAD_65/67_) in post-hatch chickens (P2–P10; *n* = 5) was determined using a specific polyclonal antibody against GAD_65/67_ (Millipore). Hatchlings were deeply anesthetized with Fatal-Plus (Vortech Pharmaceuticals) and transcardially perfused with PFA. The brains were dissected out; post-fixed in PFA for 2 h at room temperature (RT) and then O/N at 4°C; rinsed thoroughly in PBS, pH 7.4; and vibratome sliced (50 μm in thickness). The free-floating sections were rinsed in PBS. Endogenous peroxidase activity was quenched for 30 min in 3% H_2_O_2_ (in 80% methanol-PBS), and nonspecific binding sites were blocked for 2 h in 5% normal goat serum (in 0.5% Triton X-100 and PBS), followed by a block for endogenous avidin and then biotin for 15 min each (Vector Laboratories). Sections were incubated with the primary antibody [GAD_65/67_: 1:1000 in 2% normal goat serum, O/N at RT; catalog #AB1511, Millipore (RRID: AB_11210186)], followed by incubation with the biotinylated goat anti-rabbit secondary antibody (1:200; Vector Laboratories) for 2 h at RT. The signal was amplified using an avidin–biotin–horseradish peroxidase-based system (Vector Laboratories). Sections were rinsed in Tris-buffered saline, pH 7.4, prior to being reacted with DAB enhanced with osmium and allowed to air dry (O/N at RT). Sections were coverslipped using Permount (Fisher Scientific). Negative control experiments were performed with omission of the primary antibody or secondary antibody. Images were taken with a high-resolution CCD camera system (SPOT Digital Camera, Diagnostic Instruments) mounted on an Olympus Provis AX70 Microscope.

### Data analysis

The RMP was determined immediately after whole-cell mode was established. Events of spontaneous IPSCs (sIPSCs) were detected by a template function using a function for product of exponentials, *f*(*t*) = [1 − exp(−t/rise time)] × exp(−*t*/decay tau), where *t* stands for time and tau stands for time constant. The values of the parameters for the template are as follows: amplitude, −40 pA; rise time, 0.6 ms; decay tau, 10 ms; with a template baseline of 30 ms and a template length of 30 ms. These parameters were determined based on an average of visually detected synaptic events. The detection threshold is threefold the noise SD, which detects most of the events with the least number of false positives. The average of detected events for each cell was obtained using AxoGraph to measure rise time, amplitude, and decay tau. The reversal potential was determined by plotting the membrane holding potential against the peak current amplitude and calculating the *x*-intercept based on a line of best fit. Internal Cl^−^ concentration was calculated using the Nernst equation. Normalized spike probability was calculated by subtracting the number of action potentials (APs) during contralateral stimulation from the baseline number of APs and averaging this difference from a minimum of four repetitions per experimental condition.

Statistical analysis was performed with SPSS (version 23, IBM) and plotted using Igor Pro (version 6.01, WaveMetrics). Values are reported as the mean and SEM. Statistical differences were determined by two-tailed independent *t* test to compare values from medial and contralateral stimulation groups and by a one-way ANOVA for experiments with multiple drug treatment groups. Comparisons of values obtained within the same cell were made using a paired *t* test, or repeated-measures ANOVA (RM-ANOVA) when more than two measurements were made over time. The normalized spike probability for hyperpolarizing and depolarizing inhibition was compared across four different levels of inhibition by a two-way ANOVA. When significant differences were observed in an ANOVA, Fisher’s least significant difference *post hoc* analysis was conducted to determine individual group differences. For significant differences observed in a two-way ANOVA or RM-ANOVA, a Bonferroni-corrected paired comparison was conducted for individual sample comparisons. Significant differences were defined as a value of *p* < 0.05.

## Results

### Synaptic inhibition in the LLDp is largely GABAergic

In the barn owl, the LLDp contains neurons positive for GAD (Carr et al., 1989), and GABA has been proposed to be the major neurotransmitter for the inhibition (Takahashi et al., 1995). However, *in vivo* application of bicuculline, a GABA_A_ receptor antagonist, does not consistently block the inhibition produced by sound stimulation of the ipsilateral ear (Adolphs, 1993), suggesting possible involvement of other inhibitory transmitters. To determine the inhibitory neurotransmitters in this circuit of the chicken, synaptic inhibition was evoked with electrical stimulation via a bipolar concentric tungsten electrode placed directly medial to the LLD in the commissure of Probst, which contains the projecting fibers from the contralateral LLDp (Fig. 1*C*). Whole-cell recordings were performed in the presence of DNQX (50 μm, AMPAR antagonist) to isolate inhibitory responses. In most cells, evoked IPSC (eIPSC) amplitude was not affected by bath application of glycine receptor antagonist (strychnine, 1 μm), but eIPSCs were abolished by GABA_A_R antagonist application (gabazine, 10 μm; Fig. 1*D*,*E*). However, an additional glycinergic component in eIPSCs was occasionally observed (3 of 13 cells), suggesting that LLDp neurons may also receive glycinergic inhibition (Fig. 1*F*). The average amplitude for medial GABAergic eIPSCs was −453.5 ± 86.0 pA (*n* = 10; Fig. 1*G*). The average 20–80% rise time was 0.54 ± 0.07 ms (*n* = 10; Fig. 1*H*), and the decay tau was 9.3 ± 1.7 ms (*n* = 10; Fig. 1*I*). These results indicate that the major inhibitory inputs to the LLDp are GABAergic, with similar pharmacology and kinetics to the inhibitory inputs to NA neurons (Kuo et al., 2009), where the excitatory inputs to LLDp originate.

### LLDp neurons have functional GABA_A_ and glycine receptors

**Figure 2.**
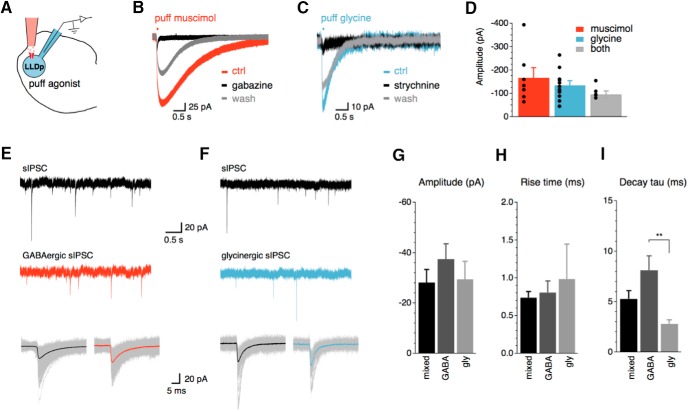
LLDp neurons have functional GABA_A_ and glycine receptors. ***A***, Schematic of the experimental setup highlighting direct puff application of receptor agonists to the recorded cell. ***B***, ***C***, In all neurons recorded, puff application of muscimol (GABA_A_ receptor agonist, 10 μm, red; ***B***) and glycine (glycine receptor agonist, 500 μm, blue; ***C***) evoked IPSCs, which were blocked by their respective antagonists, gabazine (10 μm) and strychnine (1 μm). ***D***, Amplitude of IPSCs did not significantly differ among puff application of muscimol (*n* = 7), glycine (*n* = 11), or a mixture of muscimol and glycine (*n* = 5), suggesting that GABA and glycine may interfere with each other. ***E***, ***F***, sIPSCs (top, black) were pharmacologically isolated into GABAergic sIPSCs (***E***, red) or glycinergic sIPSCs (***F***, blue) with bath application of strychnine (1 μm) or gabazine (10 μm), respectively. Averaged sIPSCs (bottom, thick lines) show distinct decay kinetics between GABAergic and glycinergic sIPSCs. ***G–I***, Population data of sIPSC amplitude, 20–80% rise time, and decay tau. Population data for decay tau (***I***) shows significant difference between GABAergic (*n* = 3) and glycinergic sIPSCs (*n* = 3; *p* = 0.008, ANOVA with *post hoc* Fisher’s exact test). For this and subsequent figures: *p < 0.05, **p < 0.01, and ***p < 0.001. ctrl, Control; gly, glycine.

To further investigate the prevalence of mixed GABAergic and glycinergic input to LLDp neurons, we used puff application (5–10 psi; duration, 25–50 ms) of GABA_A_ receptor agonist (muscimol, 10 μm), glycine receptor agonist (glycine, 0.5 mm), or a combination of both agonists, to activate the receptors and bypass the presynaptic release of transmitters (Fig. 2*A*). Puff application of muscimol (Fig. 2*B*) and glycine (Fig. 2*C*) elicited IPSCs in all cells tested, and the IPSCs were blocked by their respective antagonists, gabazine (10 μm) and strychnine (1 μm), suggesting that both GABA and glycine can affect LLDp neurons. The average amplitude of IPSCs evoked from the application of muscimol (−168.1 ± 42.4 pA, *n* = 7), glycine (−136.1 ± 19 pA, *n* = 11), and both agonists (−97.1± 14.2 pA, *n* = 5; *p* = 0.41, ANOVA) did not significantly differ (Fig. 2*D*). The response to both agonists was equal to or smaller than the response to each individual agonist, suggesting interference between the two transmitter systems (Trombley et al., 1999). The response to glycine in all cells tested is in contrast to the observation that a small portion of LLDp neurons had a glycinergic eIPSC component (Fig. 1*F*), suggesting that activation of the glycinergic component may require heightened stimulation in both temporal and frequency domains (Fischl et al., 2014) or activation of a distinct synaptic input other than the medial pathway.

Given the evidence that LLDp neurons have functional glycine receptors, we hypothesized that there should be functional glycinergic inputs as well. To test this, we recorded sIPSCs and pharmacologically blocked glycine receptors (strychnine, 1 μm) or GABA_A_ receptors (gabazine, 10 μm) to isolate the GABAergic and glycinergic sIPSCs, respectively (Fig. 2*E*,*F*). The amplitude of sIPSCs was not significantly different between groups (mixed: −28.2 ± 5.1 pA, *n* = 7; GABAergic: −37.6 ± 6.0 pA, *n* = 3; glycinergic: −29.5 ± 7.1 pA, *n* = 3; *p* = 0.57, ANOVA; Fig. 2*G*), nor was the 20–80% rise time (mixed: 0.70 ± 0.1 ms, *n* = 7; GABAergic: 0.81 ± 0.1 ms, *n* = 3; glycinergic: 0.55 ± 0.1 ms, *n* = 3; *p* = 0.16, ANOVA; Fig. 2*H*). However, the decay tau was significantly larger for GABAergic sIPSCs (8.1 ± 1.4 ms, *n* = 3; *p* = 0.008, ANOVA *post hoc* Fisher’s least significant difference) than glycinergic sIPSCs (2.8 ± 0.4 ms, *n* = 3), but was not significantly different from the mixed sIPSCs (5.4 ± 0.9 ms, *n* = 7; *p* = 0.062; Fig. 2*I*). Only three of six neurons had detectable glycinergic sIPSCs during pharmacological isolation, but GABAergic sIPSCs were always present. Together, these results support the idea that the major inhibitory inputs to the LLDp are GABAergic. Glycinergic inputs may target only a subset of LLDp neurons or be activated only under specific conditions.

### IPSCs can be evoked electrically or chemically from activation of the contralateral LLDp

**Figure 3.**
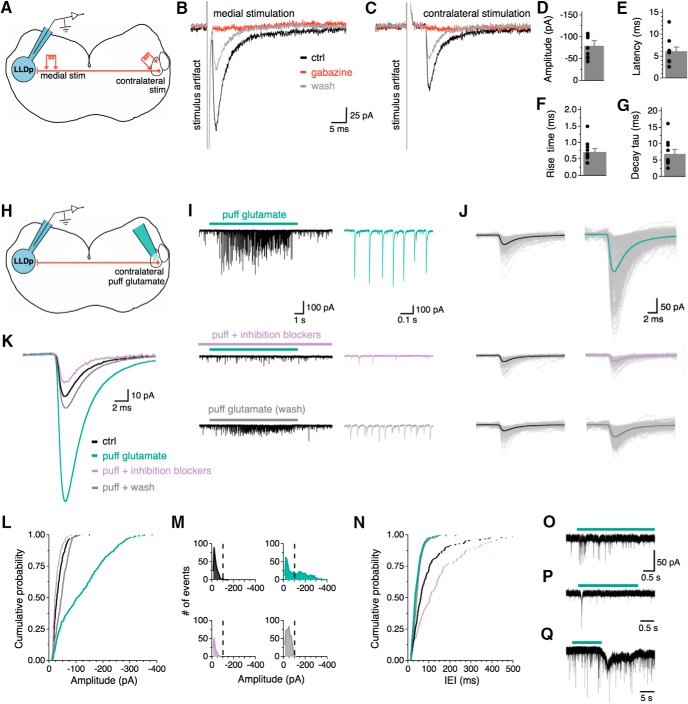
IPSCs can be evoked electrically or chemically from activation of the contralateral LLDp. ***A***, Schematic of the experimental setup for electrical activation of contralateral LLDp, highlighting ipsilateral recording site (blue), and the following two stimulating locations: medial (left) and in the contralateral LLDp (right). ***B***, ***C***, Averaged eIPSCs from medial LLDp (***B***) and contralateral LLDp (***C***) stimulation in the same neuron. Note the larger amplitude and shorter latency in the medial eIPSC. Bath application of gabazine (10 μm, red) completely abolished both medial and contralateral eIPSCs. ***D–G***, The contralateral eIPSC population data for amplitude, latency, 20–80% rise time, and decay tau (*n* = 10). ***H***, Schematic of experimental setup for chemical activation of contralateral LLDp, highlighting ipsilateral recording site (blue), and direct puff application of glutamate in the contralateral LLDp (green, right). ***I***, For a single neuron, puff application of glutamate (150 μm, 5–10 psi, 10 s) on the contralateral LLDp produced PSCs that were abolished by bath application of gabazine (10 μm) and strychnine (1 μm). Enlarged views of PSCs are shown to the right during puff glutamate (top, green), puff glutamate with inhibition blockers (middle, purple), and wash (bottom, gray). ***J***, Overlay of individual PSCs (thin lines) and their average (thick line) during control (left, black) and during glutamate puff application in the following conditions: ACSF (top), inhibition blockers (middle), and wash (bottom). ***K***, Overlay of averaged PSCs. ***L***, Cumulative probability of PSC amplitude shows that PSCs during puff glutamate (green) have larger amplitudes compared with control (black) and puff glutamate with inhibition blockers (purple) conditions in a sample cell. ***M***, Distribution of PSC amplitude shows a bimodal distribution of PSCs during glutamate application (green), with a population of events >100 pA, which is not seen in the control or during the inhibition blockers condition. ***N***, Cumulative probability of the IEI between PSCs shows a decrease in IEI during puff glutamate (green) and wash (gray) compared with control (black), and puff glutamate compared with inhibition blockers (purple). ***O–Q***, Sample traces from three individual neurons with varying degrees of responsiveness to contralateral glutamate puff (green bar). ctrl, Control.

The contralateral LLDp is believed to be the major source of inhibition in the avian ILD coding circuit, which has been supported by experiments in both owl (Manley et al., 1988; Carr et al., 1989; Takahashi and Keller, 1992; Adolphs, 1993) and chicken (Sato et al., 2010). Here we report the first direct physiological evidence of a monosynaptic connection between the LLDps *in vitro*. The medial stimulation in the commissure of Probst is likely to activate the projecting fibers from the contralateral LLDp, but it may also activate inhibitory inputs from other unknown sources. To better isolate the inhibitory inputs from the opposite LLDp, a bipolar concentric tungsten electrode was placed directly in the contralateral LLDp; a second stimulating electrode was also placed medial to the LLDp for a subset of cells to compare the evoked responses (Fig. 3*A*). Within the same cell, both medial (Fig. 3*B*) and contralateral (Fig. 3*C*) eIPSCs were not affected by AMPAR antagonist application (DNQX, 50 μm), confirming that the major projection between the LLDps is monosynaptic. If it were not monosynaptic (i.e., the inhibitory input is driven by another glutamatergic neuron located between the stimulation site and the recorded cell), the contralateral eIPSCs, but not the medial eIPSCs, would diminish upon blocking AMPARs. Activation of synaptic inputs by directly stimulating the contralateral LLDp in a 300-µm-thick slice was achieved in more than half of the cells tested [40 of 65 cells (62%)], which indicates that the fiber connections between the two LLDps in a coronal section are preserved, and they project in a generally straightforward path across the midline, consistent with the anatomical observation in barn owl (Takahashi and Keller, 1992). The lack of contralateral response in the rest of the tested cells can be explained by differences in the preservation of fibers within the slice, variations in the placement of the stimulating electrode, or perhaps differences in contralateral inputs to cells. Contralateral eIPSC amplitude (−80.3 ± 11.3, *n* = 10; Fig. 3*D*) was significantly smaller compared with medial eIPSC amplitude (−454.1 ± 86.2 pA, *n* = 10; *p* = 0.002, *t* test), reflecting the limited recruitment of afferent neurons/fibers by the contralateral stimulation. As expected from the long distance between the two LLDps (∼4 mm), the response latency was approximately fivefold longer in contralateral eIPSCs than the medial eIPSCs (contralateral: 6.2 ± 2.9 ms, *n* = 10, Fig. 3*E*; medial: 1.4 ± 0.4 ms, *n* = 10; *p* < 0.001, *t* test; data not shown). The 20–80% rise time for contralateral eIPSCs averaged 0.7 ± 0.3 ms (*n* = 10, Fig. 3*F*), and decay tau was 7.0 ± 4.3 ms (*n* = 10; Fig. 3*G*).

The establishment of the reciprocal inhibitory connection between the two LLDps is critical for understanding the avian ILD coding mechanism. To substantiate the results obtained with the contralateral electrical stimulation, we chemically activated the contralateral LLDp neurons and recorded responses in the ipsilateral LLDp (Fig. 3*H*). A pipette filled with ACSF containing 150–300 μm glutamate was placed above the contralateral LLDp for puff application (5–10 psi, 100 ms pulse, 0.5 Hz). To search for synaptic connections, the puff pipette was moved in a dorsal-to-ventral fashion while recording from a neuron in the ipsilateral LLDp. Once evoked activity was observed, glutamate was puff applied for 10 s under the following conditions: puff glutamate, puff glutamate during administration of inhibition blockers (gabazine and strychnine), and wash. Glutamate receptors were not blocked because that would disable the chemical activation of the inhibitory neurons in the contralateral LLDp. Amplitude, interevent interval (IEI), 20–80% rise time, and decay tau of postsynaptic currents (PSCs) were measured before and during glutamate application. For a single LLDp neuron with a strong response (Fig. 3*I*), the evoked PSCs were larger in amplitude (−120.6 ± 3.5 pA) than the PSCs under control (−34.4 ± 1.2 pA). The responses diminished upon puff application of glutamate during the administration of inhibition blockers (−27.0 ± 1.3 pA) and recovered partially after wash of the inhibition blockers (−46.4 ± 0.1 pA; Fig. 3*I–L*). Distributions of PSC amplitude (Fig. 3*M*) show a bimodal distribution of PSCs during glutamate puff (Fig. 3*M*, top right), with a population of PSCs >100 pA, which was not seen in the other conditions. This supports the idea that the PSCs evoked by the contralateral puff of glutamate are a separate population of synaptic events from the background activity. Additionally, the contralateral puff-evoked PSCs were abolished with the addition of the inhibition blockers (Fig. 3*M*, bottom left), supporting that the idea excitation of the contralateral LLDp gives rise to inhibitory neurotransmitter release in the ipsilateral LLDp. The average IEI of PSCs during puff glutamate application was shorter (20.1 ± 1.0 ms, Fig. 3*N*) than that of control PSCs (81.9 ± 4.4 ms) and that of PSCs during puff glutamate in the presence of inhibition blockers (67.3 ± 9.2 ms), with recovery seen during the wash condition (45.8 ± 1.1 ms). The success of finding a contralateral LLDp region to which puffed glutamate evoked PSCs in the ipsilateral recorded cell was low (4 of 19 cells), which may be a result of the small contralateral area stimulated or insufficient depolarization of contralateral LLDp neurons by puffed glutamate. Responses were most commonly seen when the glutamate application was in a region similar to that of the recorded cell (i.e., dorsal or ventral). In addition to the robust response to the contralateral glutamate puff, responses consisting of shorter bursts of PSCs or transient inward currents were also observed (Fig. 3*O–Q*). Together, these results support the idea that activation of the contralateral LLDp directly drives an inhibitory input to the other LLDp.

### Intrinsic regulation of the E_rev_ of IPSCs in LLDp neurons

**Figure 4.**
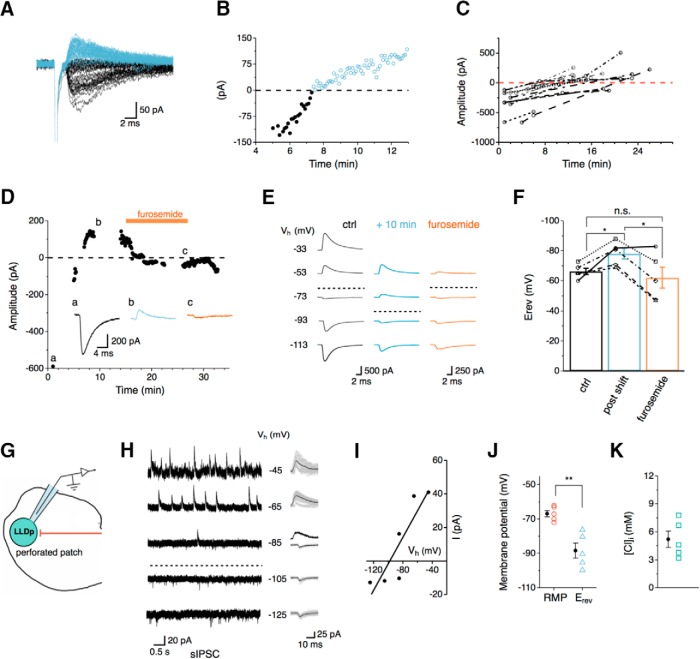
Intrinsic regulation of the E_rev_ for Cl^−^ channels in LLDp neurons. ***A***, eIPSCs from a sample neuron were inward initially (black) and shifted polarity (blue) during whole-cell recording. ***B***, The eIPSC amplitudes are plotted over time showing that the shift occurred at about 8 min after whole-cell recording began. ***C***, Population data of eIPSC amplitude over time (*n* = 16). eIPSCs were largely observed as inward currents initially, but in many cells the current became outward over time during whole-cell recordings. The shift in polarity generally occurred within 20 min. ***D***, After the eIPSC became outward, bath application of furosemide (500 μm), a KCC2 antagonist, returned the eIPSC to an inward current. Inset, eIPSC traces correspond to the following conditions: control (a, 1 min), after the polarity shift (b, 10 min), and during furosemide application (c, 28 min). ***E***, The E_rev_ during control (left), after the polarity shift (middle, +10 min), and during furosemide application (right) was determined by stepping the holding potential from −113 up to −33 mV (increment of 20 mV) during whole-cell recordings. The dashed lines approximately indicate the E_rev_. ***F***, Average E_rev_ was 11.8 mV more hyperpolarized than control after the polarity shift (*n* = 5, blue) and returned to near control levels during furosemide application (*n* = 5, orange). For the four of five cells that were affected by furosemide, the E_rev_ after the polarity shift was significantly different from control and furosemide application (*p* = 0.036, RM-ANOVA with *post hoc* Bonferroni test). ***G***, Schematic of experimental setup for gramicidin-perforated patch recording, highlighting the gramicidin-containing internal solution (with high Cl^−^ concentration, 145 mm) and native Cl^−^ concentration within the LLDp neuron. ***H***, ***I***, With gramicidin-perforated patch recording, sIPSCs were recorded under different membrane holding potentials (−125 up to −45 mV, increment of 20 mV) to determine E_rev_. Individual and averaged sIPSCs are shown to the right. ***J***, The calculated E_rev_ for sIPSCs (*n* = 5) was 21.6 mV more negative than the average RMP (*p* = 0.022, paired *t* test). ***K***, The calculated Cl^−^ concentration was relatively low (*n* = 5). ctrl, Control.

Interestingly, the IPSC E_rev_ in LLDp neurons showed time-dependent changes. Based on the Cl^−^ concentrations in the internal solution (14 mm) used for WCR and in ACSF (141 mm), the calculated reversal of IPSCs was −61 mV, such that IPSCs recorded at a holding potential of −73 mV were inward currents. However, many neurons shifted from an inward IPSC to an outward IPSC during recording (Fig. 4*A–C*). This is presumably due to a reduction of intracellular Cl^−^ concentration, suggesting that E_rev_ changed over time. The polarity shift of eIPSCs was typically observed within 20 minutes of recording (*n* = 16; Fig. 4*C*). We hypothesized that if the LLDp neurons were reducing the internal Cl^−^ concentration, it may occur through the K-Cl cotransporter KCC2. KCC2 was selected as a potential driver of the Cl^−^ concentration shift because KCC2 can maintain low intracellular Cl^−^ concentrations through Cl^−^ extrusion and can be modulated in an activity-dependent manner (for review, see Ben-Ari, 2002; Blaesse et al., 2009; Chamma et al., 2012). To test this, the IPSC E_rev_ was measured under the following conditions: inward IPSC (control); outward IPSC (after polarity shift); and after a 10 min bath application of furosemide (0.5 mm), a KCC2 antagonist. The maximal eIPSC amplitude was determined within the first 30 s after whole-cell break-in and was monitored with a single pulse (0.17 Hz) until the polarity shift occurred and became stable, at which point furosemide was bath applied (Fig. 4*D*) and eIPSCs at varying holding potentials were obtained in order to calculate E_rev_ (Fig. 4*E*,*F*). For the four of five neurons that responded to furosemide, IPSCs reversed at −68.0 ± 3.8 mV in the control condition. The eIPSCs became outward on average by 10 min after whole-cell break-in (polarity shift) and resulted in a significant hyperpolarization of E_rev_ to −77.3 ± 8.9 mV (*p* = 0.036, RM-ANOVA with *post hoc* Bonferroni test). The suppression of KCC2 activity with furosemide returned E_rev_ values to a level that did not significantly differ from baseline (−57.0 ± 12.2 mV, *p* = 0.078). This finding implicates the role of the Cl^−^ transporters in shifting IPSCs from depolarizing to hyperpolarizing when LLDp neurons were challenged with high intracellular Cl^−^ concentrations.

To determine whether depolarizing or hyperpolarizing IPSCs were more physiologic, a gramicidin-perforated patch clamp was implemented to estimate the native intracellular Cl^−^ concentration. sIPSCs were recorded while systematically changing the membrane holding potential (Fig. 4*H*). Amplitudes of the sIPSCs plotted against the membrane holding potential were fitted with a line regression and extrapolated to zero current to determine the E_rev_ (Fig. 4*I*). At a holding potential (−85 mV) close to E_rev_, both inward and outward sIPSCs were observed (Fig 4*H*), suggesting that LLDp neurons may receive inhibitory input on local compartments that have different Cl^−^ concentrations. On average, E_rev_ was significantly more hyperpolarized (−88.4 ± 4.4 mV, *n* = 5) than the RMP (−66.8 ± 1.9 mV, *n* = 5; *p* = 0.011, paired *t-*test; Fig. 4*J*). Based on the calculated E_rev_, the intracellular Cl^−^ concentration was estimated to be 5.2 ± 0.9 mm (*n* = 5). This suggests that, under resting conditions, inhibition in the LLDp is hyperpolarizing.

### Contralateral synaptic inhibition reduces firing of LLDp neurons

**Figure 5.**
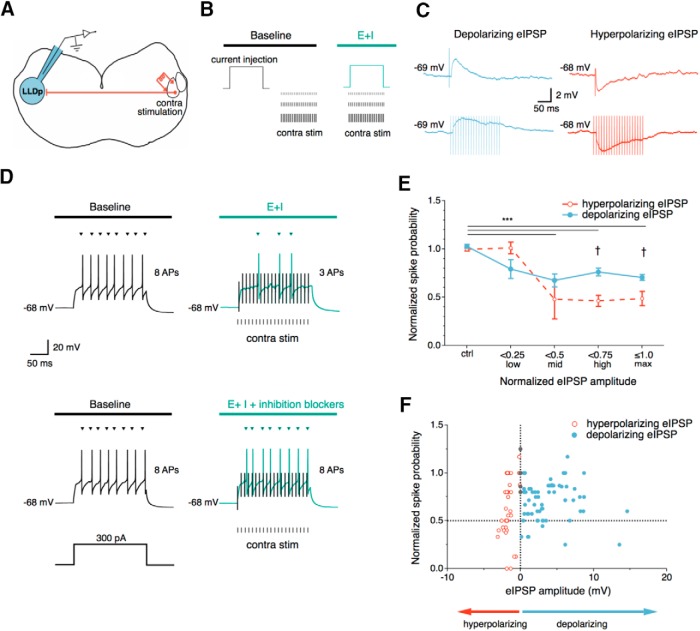
Contralateral synaptic inhibition reduces firing in LLDp neurons. ***A***, Schematic of the experimental setup, highlighting the electrical stimulation of the contralateral LLDp (right) during whole-cell current-clamp recordings. ***B***, Schematic of the current-clamp protocol used to evaluate the effects of contralateral inhibition on spiking activity. A depolarizing current step (200 ms, 100 pA above threshold) was injected into the cell body to evoke APs. This was followed by or overlapped with a contralateral electrical stimulation (100 Hz, 200 ms, 20 pulses) for the baseline condition and the experimental condition, respectively. E+I, With overlapping excitation and inhibition. The intensity of the electrical stimulation was increased in a stepwise fashion to elicit 0 to maximal eIPSP. ***C***, Example of depolarizing eIPSPs (left, blue) and hyperpolarizing eIPSPs (right, red) in response to the contralateral stimulation (top, single-pulse; bottom, 100 Hz stimulation). ***D***, The contralateral stimulation decreased the number of APs (top, right) compared with baseline (top, left). Bath application of the inhibition blockers gabazine (10 μm) and strychnine (1 μm; bottom, right) eliminated the effect. Stimulus artifacts from contralateral stimulation are truncated for clarity and shown in black. ***E***, The normalized spike probability was sensitive to both hyperpolarizing and depolarizing eIPSPs during increasing contralateral stimulation levels. Normalized spike probability was significantly reduced during mid, high, and max normalized eIPSP amplitude levels, compared with control levels (*n* = 11, *p* < 0.001, two-way ANOVA with *post hoc* Bonferroni test). Normalized spike probability was significantly lower for hyperpolarizing inhibition (*n* = 3) than depolarizing inhibition (*n* = 8) at high and max normalized eIPSP amplitudes (†*p* = 0.002). ***F***, Normalized spike probability plotted against eIPSP amplitude of both polarities. The horizontal dashed line indicates a 50% reduction of APs, and the vertical dashed line at 0 mV separates the hyperpolarizing (negative) and depolarizing (positive) eIPSP amplitudes. Contra Stim, Contralateral stimulation.

To determine how the contralateral inhibition modulates the firing of LLDp neurons, a somatic current injection (200 ms, 100 pA above threshold) was applied to elicit APs. Then, the intensity of a contralateral electrical stimulation (100 Hz, 200 ms, 20 pulses) was increased in a stepwise fashion during the current injection to elicit a range of evoked IPSP (eIPSP) amplitudes from 0 (no contralateral stimulation) to maximal eIPSP amplitude. The number of APs during the contralateral stimulation condition was compared to the baseline condition (current injection, no contralateral stimulation; Fig. 5*A*,*B*). eIPSPs were typically observed as depolarizing (*n* = 8), but could become hyperpolarizing over time (*n* = 3; Fig. 5*C*). Contralateral stimulation reduced the number of APs, and this effect was abolished by the application of inhibition blockers (Fig. *5D*). The latency of eIPSPs averaged 6.0 ± 1.4 ms and could modulate the first AP, which occurred 15.6 ± 3.6 ms after the current step onset (*n* = 11). The spike probability during contralateral stimulation was normalized to the control condition (no contralateral stimulation), and the eIPSP amplitude was normalized to the maximal eIPSP amplitude for each neuron. The normalized spike probability was compared among the following four different normalized eIPSP levels: control (no contralateral stimulation), low (normalized amplitude, <0.25), mid (normalized amplitude, ≥0.25 and <0.5), high (normalized amplitude, ≥0.5 and <0.75), and maximum (max; normalized amplitude, ≥0.75 and ≤1.0). Overall, the main effect of normalized eIPSP amplitude was significant across amplitude levels (*F*_(4,127)_ = 25.93, *p* < 0.001, two-way ANOVA with *post hoc* Bonferroni test), with normalized eIPSP amplitudes at mid levels and above differing significantly from control (control, 1.00 ± 0.03; mid, 0.58 ± 0.60, *p* < 0.001; high, 0.61 ± 0.05, *p* < 0.001; max, 0.60 ± 0.03, *p* < 0.001). The main effect of polarity was also significant (*F*_(1,127)_ = 5.9, *p* = 0.017, two-way ANOVA with *post hoc* Bonferroni test), with hyperpolarizing eIPSPs resulting in lower normalized spike probabilities at high (0.46 ± 0.06, *p* = 0.002) and max normalized eIPSP amplitudes (0.49 ± 0.06, *p* = 0.002) when compared with depolarizing eIPSPs at the respective levels (depolarizing high, 0.76 ± 0.07; depolarizing max, 0.71 ± 0.03). This suggests that as normalized eIPSP amplitude increases, hyperpolarizing inhibition reduces normalized spike probability more than depolarizing inhibition, even though both hyperpolarizing and depolarizing inhibition can reduce the normalized spike probability. Comparing the raw IPSP amplitude against the normalized spike probability (Fig. 5*F*), hyperpolarizing eIPSPs tended to be more effective at reducing spike probability than depolarizing eIPSPs, forming a contrast to nucleus magnocellularis (NM) neurons where depolarizing inhibition has a stronger suppression on cellular excitability than hyperpolarizing inhibition (Monsivais and Rubel, 2001).

### LLDp neurons have diverse cell morphology and are GAD_65/67_ positive

**Figure 6.**
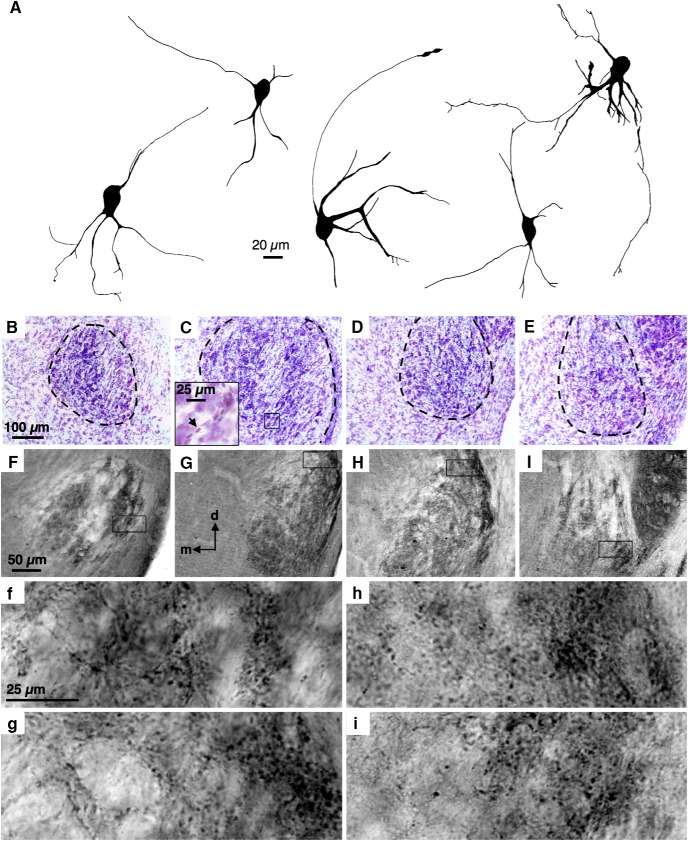
LLDp neurons have diverse cell morphology and are GAD_65/67_ positive. ***A***, Biocytin-filled LLDp neurons have diverse morphology. Most cells have large somas but exhibit different dendritic branching patterns. ***B–E***, Serial sections (50 µm in thickness) of a Nissl stain through the LLD (from left to right: caudal to rostral). LLDp neurons appear more densely distributed at caudal levels, with the number of cells becoming sparser in rostral sections. A magnified view of an oligodendrocyte (arrow) is shown in the inset (***C***). ***F–I***, Serial sections of a GAD_65/67_ stain through the LLD (left to right: caudal to rostral). Corresponding magnified view is shown below, highlighting the punctate staining of GAD-positive terminals (***f–i***). GAD_65/67_ staining highlights the segregation of the anterior and posterior LLDs, with denser staining on the posterior portion associated with ILD-coding neurons in chicken. At rostral levels, dorsal cells exhibit weaker GAD staining than ventral cells (***I***). d, dorsal; m, medial.

To support our physiological observations, we examined the morphology of biocytin-filled cells (Fig. 6*A*), the distribution of neuronal cell bodies (Nissl stain; Fig. 6*B–E*), and GAD_65/67_ immunoreactivity in the LLDp (Fig. 6*F–I*). Bioctyin-filled cells often had large somas and expressed a variety of dendritic branching patterns. Axons could often be observed projecting toward the midline (data not shown). Currently, no classification of morphological cell type has been generated for LLDp neurons; however, it is possible that morphological differences may have functional significance, such as certain classes of cells being directly responsible for the reciprocal inhibition, and other classes computing the ILD. Serial sections (50 µm) of a Nissl stain through the LLD (caudal to rostral; Fig. 6*B–E*) highlighted the large somas of LLDp neurons, which are located in the lateral portion of the LLD. The density of the large soma cells decreased toward rostral regions. Oligodendrocytes, which appear as darkly stained small cell bodies (Garman, 2011), were prominent throughout the LLDp and were often aligned along putative fiber tracts (Fig. 6*C*). In the barn owl, GABAergic neurons are dispersed throughout the LLDp, with dorsal regions having a slightly higher population of GAD-positive cells, and LLDp having more GABAergic cells and terminals than the anterior portion of the dorsal nucleus of the lateral lemniscus (LLDa; Carr et al., 1989). GAD_65/67_ immunoreactivity, visualized with DAB reaction product (Fig. 6*F–I*), was evident throughout the LLD and appeared slightly denser in the LLDp region compared with the LLDa region, with strong labeling of GAD-positive terminals (Fig. 6*F–I*). The lamina between the LLDa and LLDp was prominent through mid-level sections (Fig. 6*F–G*), and the immunoreactivity was less strong at the dorsal region of the most rostral sections (Fig. 6*I*).

## Discussion

The results of this study reveal that the synaptic inhibition in the LLDp is largely GABAergic, with the presence of functional glycine receptors in LLDp neurons. We report the first evidence of the direct activation of the LLDp driving GABAergic inhibition in the other LLDp. This reciprocal connection is monosynaptic and can dynamically inhibit spiking in the LLDp, forming the cellular and circular basis for ILD coding. The inhibition is through a conventional hyperpolarizing mechanism, which is maintained by the Cl^−^ transporter KCC2.

### Transmitter types and kinetics of inhibitory responses

In birds, the excitatory inputs from the auditory nerve bifurcate into two distinct circuits to encode ITDs and ILDs (for review, see Konishi, 2003; Ohmori, 2014). The use of GABA as the main inhibitory neurotransmitter is typical of these circuits. The reciprocal connection between the LLDps has been presumed to be GABAergic in barn owl, based on GAD immunoreactivity (Carr et al., 1989) and *in vivo* physiological experiments (Takahashi and Keller, 1992; Adolphs, 1993). Here we report the first evidence of the direct stimulation of one LLDp driving GABAergic inhibition in the opposite LLDp. We observed minimal glycinergic components in response to contralateral stimulation, possibly because the contralateral LLDp may not be a major source of glycinergic input. The superior olivary nucleus (SON) is a potential source of glycinergic input, as it has been shown to project to the LLDp in zebra finch (Wild et al., 2010), and possible SON projections to LLDp neurons in chicken have been reported (Westerberg and Schwarz, 1995). The role of glycine could be to maintain neural inhibition in the circuit if GABA is depleted (Fischl et al., 2014; Nerlich et al., 2014), or it could modulate GABAergic inhibition through cross-suppression due to changes in the Cl^−^ driving force (Grassi, 1992; Karlsson et al., 2011) or other signaling cascades (Li et al., 2003).

Of particular importance is the time course of synaptic inhibition in the ILD circuitry, because there is a short window to compare coincident excitation and inhibition during the movement of a sound source. ILD-coding neurons must be able to balance temporal aspects of the excitatory and inhibitory inputs, even though the inhibition must traverse at least one additional synapse. In the mammalian ILD circuit, the LSO receives fast, phasic inhibition that occurs rapidly enough to oppose excitation (Park et al., 1996; Tollin and Yin, 2005). The inhibition to LLDp must also transverse at least one additional synapse compared with the excitation, which imposes similar timing constraints to the mammalian circuit, suggesting that fast inhibition would be advantageous. The GABAergic IPSCs in LLDp neurons have similar kinetics to NA and NL neurons, which are relatively fast (Kuo et al., 2009). However, under high-frequency physiological synaptic inputs, temporal summation of the inhibitory responses gives rise to sustained inhibition (Fig. 5*C*; Tang and Lu, 2012). The temporally sustained inhibition could be a mechanism by which the ILD circuit overcomes the long inhibitory latency introduced by the distance between the two LLDp. In barn owl, such a role for sustained inhibition has been suggested, because the focal blockade of GABA_A_ receptors in the contralateral LLDp resulted in disinhibition of LLDp neurons across all ILDs (Adolphs, 1993), supporting the idea that tonic GABA release could serve as a compensatory mechanism for inhibition that is slower than excitation.

### Intrinsic regulation of the polarity of synaptic inhibition

The internal Cl^−^ concentration of LLDp neurons is relatively low, similar to the avian SON (Monsivais and Rubel, 2001) and mature LSO neurons (Price and Trussell, 2006), but in contrast to the high internal Cl^−^ concentrations in NM and NL (Hyson et al., 1995; Lu and Trussell, 2001; Monsivais and Rubel, 2001; Tang et al., 2009). The low Cl^−^ concentration, maintained at least in part via KCC2 activity, supports the idea that inhibition in the LLDp is hyperpolarizing. The polarity shift of the E_rev_ observed in LLDp neurons does not generally occur during WCR in other neuronal types, in which the intracellular Cl^−^ concentration is dictated by the Cl^−^ concentration in the recording electrodes (Pusch and Neher, 1988; Lu and Trussell, 2001). However, a high Cl^−^ concentration in the intracellular pipette can maximize KCC2 activity, resulting in the deviation of the measured E_rev_ from the calculated E_rev_ based on the Nernst equation (Kopp-Scheinpflug et al., 2011; Yassin et al., 2014), suggesting that the ability of LLDp neurons to reduce the relatively higher Cl^−^ concentration imposed by the intracellular pipette solution could rely on KCC2 activity. KCC2 function may also be upregulated during periods of high inhibitory input, which could occur through a multitude of mechanisms, including group I metabotropic glutamate receptors (Banke and Gegelashvili, 2008) and activation of serotonin receptors (Bos et al., 2013). Alternative explanations for the change in E_rev_ include the rundown of GABA_A_ conductances in well dialyzed neuronal compartments, which would favor conductances through GABA_A_Rs in less dialyzed areas, as well as extrusion of Cl^−^ via chloride channels such as ClC2 when the electrochemical gradient for Cl^−^ is reversed (for review, see Doyon et al., 2016). The ability to maintain low intracellular Cl^−^ concentrations in LLDp neurons may be critical for the maintenance of inhibitory efficacy. Although depolarizing inhibition could also reduce spike probability, it tended to be less effective than hyperpolarizing inhibition and introduced large fluctuations in the membrane potential, which could degrade the encoding of ILDs.

### Avian ILD circuit and hypothetical models

**Figure 7.**
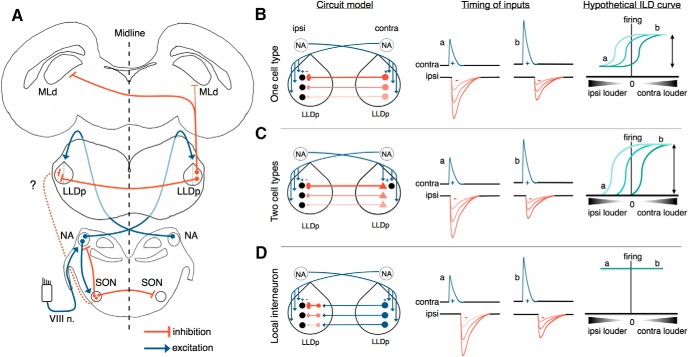
Avian ILD circuit and theoretical models for ILD coding in LLDp. ***A***. LLDp neurons receive excitatory input from the contralateral cochlear NA and inhibitory input from the contralateral LLDp, and may also receive inhibitory input from the SON, which is driven by excitatory inputs from the ipsilateral NA. LLDp neurons predominantly send inhibitory projections contralaterally to the mesencephalicus lateralis, pars dorsalis (MLd), the auditory midbrain (Wild et al., 2010). ***B–D***, Models of the origin of reciprocal inhibition for ILD coding in the LLDp. The reciprocal inhibition could arise from a single cell type that both encodes ILD and inhibits the contralateral LLDp (***B***); two cell types, one of which encodes the ILD (black circle) and another cell type that is specialized for fast synaptic transmission and provides the reciprocal inhibition (red triangles; ***C***); or local interneurons that convert excitation into inhibition (***D***). In all three hypothetical circuits, a dorsal–ventral gradient of inhibition, but not excitation, could provide a topographic readout of ILD to create a space map of sound location in the MLd. Hypothetical schematics of the timing of synaptic inputs (middle column) for EPSPs (blue) and IPSPs (red) show the timing and amplitude for each model when ipsilateral sounds are louder (a) and when contralateral sounds are louder (b). Hypothetical ILD curves (right column) based on the respective model circuit and relative timing of excitation and inhibition. The model in panel C (two cell types) may offer a full-range dynamic coding of ILD (for details, see Discussion). VIII n., 8th nerve; contra, contralateral; ipsi, ipsilateral.

We propose that the inhibition for ILD coding in the LLDp arises directly and primarily from the contralateral LLDp (Fig. 7*A*), through either the same neurons that encode ILD (one cell-type model; Fig. 7*B*) or from a second population of neurons in the LLDp (two cell-type model; Fig. 7*C*), resulting in only one additional synapse in the inhibitory pathway compared with the excitatory pathway. For the same neurons to both encode ILD and provide the reciprocal inhibition to contralateral LLDp neurons, IPSPs are likely to overlap with EPSPs, but may be delayed enough to not completely suppress firing and therefore be unable to effectively encode the entire range of ILDs (Fig. 7*B*). A potential solution to this is a tonic inhibition, which could shift the baseline firing down, as shown in a subset of barn owl LLDp neurons (Adolphs, 1993). An alternative solution could be to have two separate principal cell types in the LLDp (Fig. 7*C*), with one population specializing in comparing excitation and inhibition to encode the ILD, and another population rapidly relaying the inhibition to the contralateral LLDp, further narrowing the timing differences, allowing for the full dynamic range of ILDs to be encoded. The requirement for fast arrival of the contralateral inhibition could occur through modifications of axon diameters and myelination, as in the ITD pathway (Seidl et al., 2010, 2014; Ford et al., 2015). Our data support the idea that inhibition in the LLDp is unlikely to arise from inhibitory interneurons that convert excitation from the contralateral LLDp to inhibition (Fig. 7*D*), because the contralateral eIPSCs persisted in the presence of excitation blockers (DNQX, APV). Local inhibitory interneurons may exist in the LLDp and contribute to ILD encoding, but it is unlikely that interneurons drive the inhibition underlying the ILD computation.

In the hypothetical models, a dorsoventral gradient of inhibition, but not of excitation, could provide a topographic readout of ILD to create a space map of sound location in the auditory midbrain. In barn owl LLDp, the tuning of sound frequency is mapped in a rostrocaudal fashion, and the reciprocal LLDp connection preserves this tonotopy (Moiseff and Konishi, 1983; Manley et al., 1988). The ability to evoke IPSCs from contralateral stimulation in a coronal slice supports a rostrocaudal distribution of reciprocal projecting fibers in the chicken LLDp. Additionally, GAD_65/67_ staining was weaker at dorsal regions (Fig. 6*I*), which contrasts with the lower prevalence of GAD-positive cells in the ventral LLDp in barn owl, allowing for the possibility of an inhibitory gradient in the chicken LLDp, with opposite direction in terms of distribution to that in barn owl (Manley et al., 1988; Carr et al., 1989).

In summary, as in the ILD circuit in mammals, the avian ILD circuit is constrained by the arrival time of excitatory and inhibitory inputs to LLDp neurons due to the latency introduced by the longer inhibitory pathway. The robust monosynaptic inhibitory connection between the two LLDps helps to minimize the detrimental effect of this latency on synaptic integration. However, the ILD coding in LLDp is also constrained by the slower GABAergic inhibition that dominates the avian auditory brainstem. Therefore, the LLDp may be better adapted for the use of tonic inhibition for encoding ILD, allowing for a larger window in which excitation and inhibition can interact, much like the mammalian dorsal nucleus of the lateral lemniscus (Ammer et al., 2012) and in contrast to the mammalian LSO. Additionally, the possibility exists for modulation of the GABAergic inhibition via glycine, which could further compensate for the relatively delayed GABAergic input. Finally, LLDp neurons are well suited to maintain a low intracellular Cl^−^ concentration and hyperpolarizing inhibition, due at least in part to strong KCC2 activity, which highlights the different strategies used by the avian ILD and ITD circuits.

## Synthesis

Reviewing Editor: Marlene Bartos, University of Freiburg Decisions are customarily a result of the Reviewing Editor and the peer reviewers coming together and discussing their recommendations until a consensus is reached. When revisions are invited, a fact-based synthesis statement explaining their decision and outlining what is needed to prepare a revision will be listed below. The following reviewer(s) agreed to reveal their identity: Catherine Carr, Richard Hyson

Both reviewers point to the fact that this study is of high relevant to sound localization in birds, in particular to interactions between inhibition and excitation in response to binaural sound level differences. Moreover, the study provides some nice physiological confirmation of long-standing assumptions about the ILD pathway in birds. Therefore, only few minimal changes are required.

Reviewer 1:

Review of Synaptic Inhibition in Avian Interaural Level Difference Sound Localizing Neurons. This mss shows that IE interactions in the chicken dorsal nucleus of the lateral lemniscus mediate sensitivity to interaural level differences. This is a finding of broad relevance to studies of sound localization, since it reveals interactions between inhibition and excitation in response to binaural sound level differences. In the lemniscal nucleus, inhibition appears to be mediated by GABAA receptors, with a strong point being the demonstration that gramicidin perforated patch recordings show that the IPSPS are hyperpolarizing. The paper (revised from a previous submission) is reasonably well written, and the statistics are appropriate, although values of N are small in several cases (ie line 387). The experimental analysis of putative glycinergic effects is weak, but this does not detract from the main message of the paper. There is a paper that would be slightly relevant: Code, R. A., & Rubel, E. W. (1989). Glycine-immunoreactivity in the auditory brain stem of the chick. Hearing Research, 40(1-2), 167-172.

Minor comments

Line 72 - Sullivan and Konishi seems an odd citation

Line 75 - cite Manley here

line 241 - suspected as could be "proposed to be"

Line 354 - IEI - define here, not later

Line 363 - contralateral puff glutamate should be contralateral glutamate puff

Reviewer 2:

The authors have done a nice job addressing the criticisms of my earlier review. This includes eliminating some problematic timing data, re-running immunohistochemistry to show greater specificity, providing more detail on some of the physiological findings and rewriting various aspects of the paper. They toned down some of the potential over interpretations in the previous version. I find nothing further to request them to correct.
